# Case of Double Consciousness Connected with Hysteria

**Published:** 1849-07-01

**Authors:** T. Ogier Ward


### CASE OF DOUBLE CONSCIOUSNESS CONNECTED WITH HYSTERIA.

BY T. OGIER WARD, M.D. OXON.

As but few cases are on record of that curious form of mental disorder, termed "double consciousness," I am induced to submit to you, for insertion in the *Psychological Journal*, the following one, which, though it does not present the disease in its most intense form, and is therefore less interesting as a simple psychological phenomenon, is perhaps rendered more useful in a practical point of view, by its evident connexion with the approach and establishment of the menses, and its having been in some degree influenced by treatment.

Mary Parker, aged 13, April, 1836. Had measles at the age of seven, and has ever since been subject to cough, and pain in the side. About a year ago, while in the country for the benefit of her health, she was seized one evening with rigors, followed by an epileptic attack, in which she struggled violently, and attempted to scratch and bite the bystanders. She was not insensible, but delirious, and frequently came to herself for a few minutes. She was bled, but this only seemed to increase her struggles. She was brought home in a state of almost syncope on the fourth day; and the attacks came on at intervals for a fortnight afterwards. At the end of this time, they became much worse, sometimes assuming a cataleptic character, when the arms were extended and perfectly rigid, and the jaws clenched. At other times, she struggled greatly, and then the abdomen became much swelled. In neither kind of fit was she entirely unconscious, nor did she change colour in the face, though her appearance was wild. Her disposition was altered, for she grew excessively mischievous between the attacks, climbing over walls, &c., and beating the other children. When a paroxysm came on, she generally called her mother, and then began to struggle, and became half insensible. This state of things lasted about three months, when she partially recovered, having fits only at intervals. She then went into the country for six months, during which time she had only four or five fits, but they returned in the wagon in which her mother brought her home.

April 1st, 1836.—Saw her for the first time at the dispensary, when she presented the following symptoms: complained of headache, and pain in the stomach and left side, increased on pressure; pulse natural; tongue clean and moist, though rather white; appetite bad; bowels, which had been rather relaxed, were now confined; the glands down the neck were enlarged; hacking cough without expectoration; tenderness of spine on pressure; burning pain at the back of the head previous to the fits. I ordered eight leeches to the side, and a blister to the spine. Ferri carb. c. rheo., ter die. The leeches seemed to produce a little delirium, which continued at intervals till April 3rd, during which time she said persons were coming to take her away. On that day, she complained of intense headache till the afternoon, when she burst out crying, and seemed much alarmed at every one who approached her. From this state she passed into the opposite one of laughing and attempting to strike them. I now saw her at home, when I found her lying in bed, laughing, and half-naked. She replied very saucily to all my questions, and did not recognise me, though I had attended her brothers at home a short time previously. Complained of no pain, and denied she had ever had any; ate voraciously, which was always the case during the delirium; the bowels were confined, and her cheeks flushed; but, upon the whole, her appearance was healthy. She exhibited no signs of approaching puberty. I ordered a cold shower-bath daily. Mist. ferri c. decoct. aloes co., and tinct. assæ.

April 14th.—She has continued, since my last report, to alternate between delirium and sound state of mind. When in the former condition, she has lately become more tractable, and, as she then appears to be endued with almost preternatural strength, I have directed her mother to employ her in domestic labours, and in bringing water from a canal close by, which seems to have but little effect in inducing fatigue. But every day she has a half-fainting fit, during which she complains of her head, side, and stomach, and is very low. She then either gradually recovers her senses, or else bursts out laughing, and relapses suddenly into the previous state of delirium. Just before this change, she puts her hand to her head as if in pain.

Double consciousness is now established, for while delirious, she has little or no recollection of persons she has seen, or events which have occurred during the state of sanity, nor does she complain of any bodily pain or suffering. In the opposite state, on the contrary, she is extremely depressed, incompetent to any exertion, complains of pain in her head, side, and stomach, and is equally forgetful of all that has passed during the delirium.

April 19th.—Two days ago she became sensible, and has continued so with the exception of some fits of the epileptic kind, during which she does not change colour, nor entirely lose consciousness, but groans and struggles hard. Yesterday she nearly fainted while under the shower-bath, and to-day she is very low, and complains of pain in the head and stomach, but not in the side. Pulse 100. She feels her head much worse while lying down, but notwithstanding this symptom of congestion, I have ordered stimulants and antispasmodics.

May 2nd.—She gradually improved under the above treatment (the maniacal attacks being less durable, and the intermissions longer, and attended with fewer complaints of pain and feebleness) till April 24th, when the mania returned with all its violence, and without intermission. The pulse was strong and frequent, and a new symptom now arose. Frequently in the course of the day her legs became suddenly stiff, and she rolled about the floor in an hysterical attack, from which she as suddenly recovered, but not to perfect consciousness.

Four days ago I found her quite unmanageable, biting and scratching all who came near her, impatient of control, and, if not prevented, butting her head against the wall, or the head of the bed. I ordered her a strong solution of tart. antim., to be repeated till full vomiting was induced. This calmed her much. Yesterday she had an attack of semi-syncope, and to-day she is quiet, though still in the maniacal state. I ordered a tonic pill, composed of quin. sulph., ferri sulph., and pulv. capsici, āā gr. i., and cold applications to the head, which was to be shaved.

May 12th.—She has been rather better lately, and was sensible the whole of last night, though much depressed, and complaining of pain in her head. The period of her dispensary note having expired, I have directed her to discontinue medicine for some time.

She perfectly recovered some months afterwards, when the menses became established.

*Observations.*—The cases of double consciousness, hitherto published, have mostly occurred in young females in whom the uterine functions were disturbed, or, if in the male sex, where the nervous system had been weakened by excesses, terror, or other cerebral excitement; and they have been classed by systematic writers with epilepsy, catalepsy, somnambulism, ecstasis, or other anomalous affections.

The case related in "Mayo's Physiology," p. 195, resembles that under consideration in the patient feeling quite well, and ceasing to suffer from headache on the access of the paroxysm. Another case in which the patient's symptoms still more closely resembled the present one, is mentioned under the head of Somnambulism, in the "Cyclopædia of Medicine." In other instances, on the contrary, the patient only complained during the access, as in that mentioned by Dr. Abercrombie. The third alone of the three cases, in the first vol. of "Silliman's Journal," seems analogous to the present, inasmuch as the paroxysms were numerous; the two others being instances of long-continued delirium, from which the patients, on their recovery, resumed the train of thought that had occupied their minds just before the access, all that had passed during the interval having passed from their memory, a phenomenon of no very unusual occurrence in acute mania, and almost universally the case in the delirium of fever, and of mania â potu. The state of double consciousness was not as complete in this case as in some of the above, where there was a perfect oblivion of all antecedent circumstances; but it was sufficiently well marked to satisfy every witness how decided was the distinction between the two states of mind, which seemed to have no ideas in common beyond the existence of objects with which the patient was in constant relation. Thus, neither the house nor her friends seemed strange to her when under the influence of the disease; but other persons, less usually seen, failed to be recognised. I have not given any detailed account of the psychical phenomena presented during the maniacal attacks, because they were not of a remarkable character. Except while suffering from the epileptic or hysteric paroxysms, she took notice of what was passing around her; had no perversions of the senses, nor any hallucinations; and but from the change in her manner, which was pert and forward, and in her disposition, which became passionate, mischievous, and vindictive, and from a peculiar expression of her features, her condition would hardly have attracted any attention. Indeed, I sometimes had to inquire her state from her friends to be certain whether she were in the maniacal state or not. The fits themselves, whether epileptic or hysterical, did not differ from the ordinary forms of those diseases, except that while suffering from them, she never entirely lost her consciousness, notwithstanding that on the cessation of these fits, and her return to a sane state of mind, she retained no recollection of them, or of anything that had taken place during their continuance.

With regard to the nature of this malady, Dr. Prichard states that, though most of the cases hitherto observed in females were connected with epilepsy, yet they had an hysteric origin; and from a review of the phenomena of the present case, its spontaneous cure upon the establishment of the catemenia seems to be the most certain guide we can take in investigating its pathology; though the length of time the complaint existed (eighteen months), and the apparently complete absence of any marks of puberty during the whole period, would rather induce us to refer the origin of the disease to some previous irritation, particularly as she had never been in good health since she had the measles at seven years of age.

The first attack began with rigors, followed by an epileptic fit, to judge from the struggles of the patient; but as she did not entirely lose her consciousness, but was alternately delirious and sensible, the attack seems to have partaken more of an hysteric than of an epileptic form. The effect of the bleeding in increasing the struggles, and producing a tendency to syncope, would also bear out this view; for though bleeding is rarely used in the systematic treatment of epilepsy, yet it is generally serviceable in controlling the violence of the paroxysms, as has often been remarked, when by accident the patients have wounded themselves so as to lose a quantity of blood during a fit. In hysteria, however, the treatment is ordinarily quite opposed to bloodletting, (which has a tendency to exasperate the disease by increasing the debility, and with it the nervous susceptibility of the patient,) except in certain cases where there is evidence of some local congestion. Judged, then, by the effect of treatment, this case seems to have been hysterical ab initio, and if hysterical, dependent, therefore, upon the state of the generative system, though there existed no signs of approaching puberty. But as it is well known that menstruation may precede the other appearances of puberty, there is nothing very unreasonable in supposing that in a delicate susceptible young person, the earliest change in the condition of the generative organs might be attended with the same phenomena of convulsive action as are often observed in other cases of hysteria, where, though the external signs of puberty are present, the conclusive proof of its establishment is still wanting by the absence of menstruation. In regard to this subject, I regret that I did not make particular inquiry into the moral habits of my patient; but whatever they may have been, (and from her modest demeanour, when well, I am disposed to think favourably of them,) no information that I could have gained would have invalidated the argument for an hysteric origin of her complaint, but would have tended to confirm it.

The occurrence of tympanitis and catalepsy during the course of the disease favours this view of its nature; and the pain at the back of the head, and the tenderness of the spine, are well-known symptoms of hysterical affections, independently of the support they derive from the phrenological theory that assigns to the cerebellum the office of presiding over the generative functions, and presumes that organ to participate, by reflected sensibility, in the derangement of the sexual organs. Indeed, the first accessions of the state of double consciousness in this case were considered to be merely phases of hysteria; and it was not till after their repetition, and a close observation of their phenomena, that this state was ascertained to exist.

A certain connected train of ideas, but totally foreign to the ordinary thoughts and feelings of the patient, however, is often observed in other disorders as well as in hysteria; in delirium, for instance, particularly in delirium-tremens, and that attending typhus fever, of which ideas not a trace remains in the memory after recovery; yet such cases are not thought to be extraordinary, but merely transient disturbances of the mind—the consequences of obvious irritation of the nervous centres, which cease with the removal of their causes, like the passing off of the stupefaction of drunkenness. It is only when these phenomena are frequently repeated, or become of a permanent character, that we are struck with surprise that the mind can be almost at the same time in a sound or unsound state; and that ideas that arise in it, or impressions received by it, in either condition are only retained by it during the continuance, and on the recurrence, of that same condition, though this recurrence may not take place till after an interval of some years, when the delirium returns with all its delusions, or the reason resumes its sway, as though there had never been any interruption of its influence.

To those who have witnessed them, the phenomena of mesmerism will doubtless here suggest themselves as being analogous, if not almost identical, with this condition; but as I do not wish to say more upon a subject in regard to which there is so much diversity of opinion in the profession, I will proceed to point out the relation of double consciousness to other states of the mind, respecting which there can be no dispute. Viewed physiologically, double consciousness is connected most closely with dreaming and somnambulism, though the latter is in many instances a pathological condition; but as neither of these phases of the mind has had much light thrown upon it, even by all the improvements and discoveries of modern times, I think it better to point out the relations of the subject before us to certain disordered states of the sensorium, of which, though their proximate cause be equally unknown as that of the former, yet the exciting causes are sufficiently evident in many, and even in most cases, to admit of their treatment being undertaken with well-founded expectations of success.

We have already noticed hysteria, and the delirium of fever and mania â potu, as often exhibiting double consciousness in the oblivion of facts connected with the paroxysms; but these diseases, before they obtain this height, frequently pass through a state that is, in fact, a form of double consciousness, in which the patients still retain enough reason and reflection to know that their minds are disordered, but not enough to control the new set of feelings and ideas that are continually arising in them. The commonest form of this condition is exhibited in drunkenness, in which at one moment extravagant acts are committed, and all kinds of nonsense are uttered, and in the next the patient will deplore his folly, and put a sufficient restraint upon himself to pass for a sober person, if need be. So, also, the first abnormal ideas and feelings of hysteria may be controlled with success, but if yielded to, they will carry the patient on to the wildest extravagances and bursts of passion. A like condition often occurs in monomania and moral insanity, particularly in that form which leads to the perpetration of horrid crimes, from which the natural disposition of the patient revolts. Such cases have been adduced by Dr. Wigan, in his work on the "Duality of the Mind," in proof that the brain is not only anatomically, but psychologically a double organ, of which one hemisphere is liable to have its functions disordered, so as sometimes to predominate over, and sometimes to be subordinate to, the other healthy portion.

The increase in the strength of Mary Parker during the maniacal state is not an uncommon thing in hysterical patients, whose vigour at other times seems to be quite exhausted, and also in the last delirious struggles of mania and typhus, and is explicable by the increased irritability of the muscular system, induced by the excited state of the brain. Her insensibility to her ordinary pains and sufferings is not at all an unusual symptom in the insane, and depended upon the same condition of the sensorium, whatever it was, that produced the maniacal state. Analogous effects are now well known to be produced, without abolition of the other faculties, by anæsthetic agents, and even by drunkenness occasionally.

The change in her disposition, from bashful timidity to audacity and impertinence, must be regarded as identical with the violence and mischievous tricks of maniacs, who thereby clearly exhibit (in an exaggerated degree, however) what are the impulses of our nature when uncontrolled by reason and religion.

